# Persistent Left Superior Vena Cava with Absent Right Superior Vena Cava and Discrete Subaortic Stenosis Diagnosed in a Patient with Sick Sinus Syndrome: A Case Report and Brief Review of the Literature

**DOI:** 10.3390/diagnostics10100847

**Published:** 2020-10-19

**Authors:** Irina Demșa, Daniela Crișu, Cristian Mihai Ștefan Haba, Andreea Maria Ursaru, Vlad-Adrian Afrăsânie, Irina Iuliana Costache, Antoniu Octavian Petriș, Dan Nicolae Tesloianu

**Affiliations:** 1 Department of Cardiology, Emergency Clinical Hospital “Sf. Spiridon”, Bd. Independenței nr. 1, 700111 Iași, Romania; irina-demsa@email.umfiasi.ro (I.D.); mihai.haba@umfiasi.ro (C.M.Ș.H.); andreea_ursaru@umfiasi.ro (A.M.U.); irina.costache@umfiasi.ro (I.I.C.); antoniu.petris@umfiasi.ro (A.O.P.); dan.tesloianu@umfiasi.ro (D.N.T.); 2 Department of Internal Medicine, “Grigore.T. Popa” University of Medicine and Pharmacy, str. Universitatii nr. 16, 700083 Iași, Romania; 3 Department of Medical Oncology, “Grigore.T. Popa” University of Medicine and Pharmacy, str. Universitatii nr. 16, 700083 Iași, Romania; vlad-adrian-afrasanie@email.umfiasi.ro

**Keywords:** persistent left superior vena cava, absent right superior vena cava, dilated coronary sinus, pacemaker implantation, discrete subaortic stenosis

## Abstract

A persistent left superior vena cava (PLSVC) is the most frequent anomaly of the venous drainage system. While both a right and left superior vena cava (SVC) are usually present, a unique, left-sided SVC, also known as an isolated PLSVC, accounts for only 10–20% of cases. It is frequently associated with arrhythmias and other congenital cardiac anomalies. Though it is usually an asymptomatic condition, it may pose significant problems whenever central venous access is needed. We report a case of an isolated PLSVC that was diagnosed incidentally during pacemaker implantation for sinus node dysfunction. The venous anomaly was associated with subvalvular aortic stenosis determined by a subaortic membrane; this particular association of congenital cardiovascular anomalies is a rare finding, with only a few cases reported in the literature. We aim to highlight the clinical and practical implications of this condition, as well as to discuss the embryonic development and diagnostic methods of this congenital defect.

## 1. Introduction

A persistent left superior vena cava (PLSVC), which results from the failure of obliteration of the anterior left cardinal vein during embryonic development, is the most frequent anomaly of the venous drainage system [1]. It is found in 0.5–2% of the general population, and its incidence increases up to 10% in patients with congenital heart disease [2]. In the majority of cases, a superior vena cava (SVC) normally positioned on the right side is also present; however, in 10–20% of cases, the former is absent, a condition that is also described as an isolated PLSVC. This variant of a unique, left-sided vena cava is a significantly rare congenital anomaly, with previous studies reporting an incidence of 0.07–0.13% in the population with congenital heart disease and of 0.15% in patients who required an implantable cardiac device [3,4].

One of the most important characteristics of an isolated PLSVC is its frequent association with other congenital cardiovascular anomalies, the most frequently described being an atrial septal defect, a ventricular septal defect, a bicuspid aortic valve, and the coarctation of the aorta. However, discrete subaortic stenosis accompanying an isolated PLSVC is a rare finding, with only a few cases reported in the literature [3].

In most instances, a PLSVC opens in the right atrium through the coronary sinus without any hemodynamic impact or clinical expression. However, it has an increased arrhythmic risk due to the intrinsic dysfunction of the conduction system, and patients may present with arrhythmias and conduction disturbances. Moreover, a PLSVC can cause significant problems when central venous access is required, and it is frequently diagnosed incidentally during the placement of central venous catheters or cardiac-implantable electronic devices [4,5].

We report a very rare case of an isolated PLSVC associated with discrete subaortic stenosis that was diagnosed incidentally during pacemaker implantation. We review the embryonic basis, analyze the possible relationship between these congenital anomalies, and underline the practical implications of a PLSVC, aiming to offer a better understanding of this benign but otherwise important venous anomaly.

## 2. Case Report

**Figure 1:**
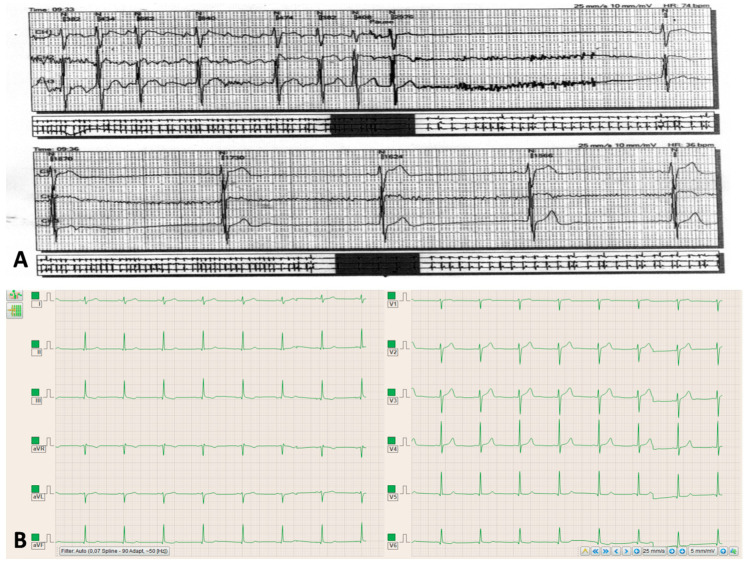
(**A**) Electrocardiographic Holter monitoring showing the tachycardia–bradycardia syndrome. (**B**) Electrocardiogram showing the sinus rhythm, benign early repolarization pattern, the increased voltage of the QRS complex, and subtle secondary repolarization changes in lead V6 and the inferior leads.

A 52-year-old male patient without a significant medical history was admitted to a local hospital reporting palpitations, lightheadedness, and presyncope symptomatology that firstly occurred one week before his admission. The electrocardiogram (ECG) demonstrated tachycardia–bradycardia syndrome, and the 24-hour ECG Holter monitoring revealed a sinus rhythm, with frequent episodes of paroxistic atrial fibrillation, a prolonged sinus pause of 2.97 seconds following the termination of tachycardia, and then the restoration of a sinus rhythm with severe sinus bradycardia before reaching the baseline heart rate of 55 beats per minute (Figure 1A).

Considering the definite correlation between the symptoms and the electrocardiographic findings, the diagnosis was of sinus node disease, brady–tachy form, with symptomatic intermittent sinus bradycardia, prolonged sinus pauses, and a Class IB indication for cardiac pacing [6]. The patient was therefore referred to our cardiology clinic for a dual-chamber, permanent pacemaker implantation.

**Figure 2:**
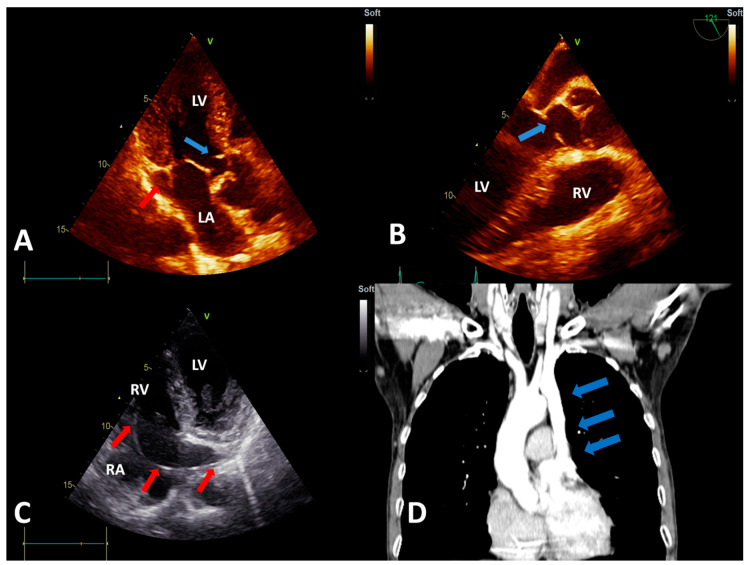
(**A**) Transthoracic echocardiography (TTE), apical 3-chamber view showing the subaortic membrane (blue arrow) and the dilated coronary sinus (red arrow). (**B**) Transesophageal echocardiography view showing the subaortic membrane (blue arrow). (**C**) TTE, modified apical 4-chamber view showing the pacemaker lead (red arrows) entering the right atrium via the dilated coronary sinus (visualized in longitudinal section). (**D**) Computed tomography angiography showing the persistent left superior vena cava (blue arrows), with an absent right superior vena cava. Abbreviations: LA, left atrium; LV, left ventricle; RA, right atrium; and RV, right ventricle.

The physical examination revealed a 4/6 systolic murmur with a maximum of intensity on the left sternal border. No diastolic murmur was present. The ECG performed in our clinic demonstrated a sinus rhythm, a benign early repolarization pattern, and an increased voltage of the QRS complex, which was interpreted as a normal feature considering the physical constitution of the patient, who was very tall and thin. However, subtle secondary repolarization changes in lead V6 and the inferior leads could suggest an early stage of left ventricle hypertrophy (Figure 1B). Transthoracic echocardiography showed a subaortic thin membrane located at 9 mm below the aortic valve (Figure 2A). The subaortic membrane caused moderate subaortic stenosis with a mean gradient of 37.3 mmHg and mild aortic regurgitation (see Supplementary Figure S1; Video S1 and S2). The cardiac cavities dimensions were normal, and the left ventricle had normal contractility and systolic function (See Supplementary Video S3). No other abnormalities were initially observed. Transesophageal echocardiography offered a better visualization of the subaortic membrane and demonstrated the systolic fluttering of the aortic cusps (Figure 2B; see Supplementary Video S4). Transesophageal echocardiography further excluded the presence of a possibly associated atrial or ventricular septal defect.

Left subclavian access was initially used, but fluoroscopic evaluation revealed that the guidewire had an abnormal trajectory in the left paravertebral region, which raised the suspicion of a venous anomaly. Therefore, venography was performed and demonstrated a PLSVC draining in the right atrium via the coronary sinus but without the possibility to clearly assess the status of the right SVC (see Supplementary Video S5). Because the team encountered difficulties in advancing the lead, they switched to the right side. Surprisingly, the venography performed from the right subclavian access demonstrated the absence of the SVC on the right side (see Supplementary Video S6). Though demanding, the ventricular electrode was eventually advanced through the left SVC and the coronary sinus. After entering the right atrium, it formed a loop in the atrial cavity in order to facilitate the passage through the tricuspid orifice. The electrode was then actively fixed in the right ventricle (see Supplementary Figure S2). However, the right atrial lead could not be implanted due to the difficult access to the right atrial appendage and the risk of the destabilization of the ventricular lead position. Postprocedural careful transthoracic echocardiography revealed a dilated coronary sinus that measured 24/26 mm and contained the pacemaker lead (Figure 2C). The flow through the coronary sinus was normal and unimpaired by the pacemaker lead. Subsequently, computed tomography angiography excluded other venous or cardiac anomalies (Figure 2D).

After cardiac pacing, the patient became asymptomatic with a normal exercise tolerance. Considering the absence of symptoms, with only moderate subaortic obstruction and mild aortic regurgitation, there was no strong recommendation for the surgical removal of the subaortic membrane according to the European Guidelines for the management of grown-up congenital heart disease [7]. Furthermore, the patient refused to take heart surgery into consideration. He was discharged with the recommendation to follow beta-blocker and direct oral anticoagulant therapy, considering the high risk of the reoccurrence of the supraventricular tachyarrhythmia. A medical reevaluation was performed after one and six months following the pacemaker implantation, and it revealed the absence of symptoms, adequate ventricular pacing, and the stationary echocardiographic parameters of the subaortic stenosis. Further reassessment is expected, close monitoring being of foremost importance to identify the optimal moment for the surgical intervention.

This research was performed in accordance with the Declaration of Helsinki of 1975 and revised in 2013. After extensive consultation, the patient gave verbal and written informed consent and fully authorized the authors to use his medical data for research purposes, as stated in the “Patient Informed Consent” (Order 1410/2016, issued by the Romanian Ministry of Health), signed by the patient.

## 3. Discussion

We aimed to present this case due to the rarity of the association between an isolated PLSVC and discrete subaortic stenosis, as well as due to the various implications and learning points that derive from it. This was the first case of an isolated PLSVC diagnosed in our center during an eight-year experience in cardiac device placement where 2834 procedures had been performed. Biffi et al. reported only two cases of an isolated PLSVC (0.15%) from a total of 1254 patients that required a pacemaker or a cardioverter-deffibrilator implantation during a 10-year period [4]. In a review of all the reported cases of isolated PLSVC from the literature published by Bartram et al. in 1997, subaortic stenosis was present only in three patients from a total of 121 cases of an isolated PLSVC reported by that time [3]. Another comprehensive study that evaluated the progression of discrete subaortic stenosis in adults did not describe any case of an associated isolated PLSVC [8]. To the best of our knowledge, there has only been one further case reported with this association of anomalies [9]. The other published cases of discrete subaortic stenosis and a left-sided vena cava do not offer details about the status of the right-sided vena cava, and several include complex malformative syndromes [10,11,12,13,14]. The purpose of the following discussion is to address the main characteristics of this venous anomaly.

### 3.1. Embryology and Anatomic Variants

**Figure 3:**
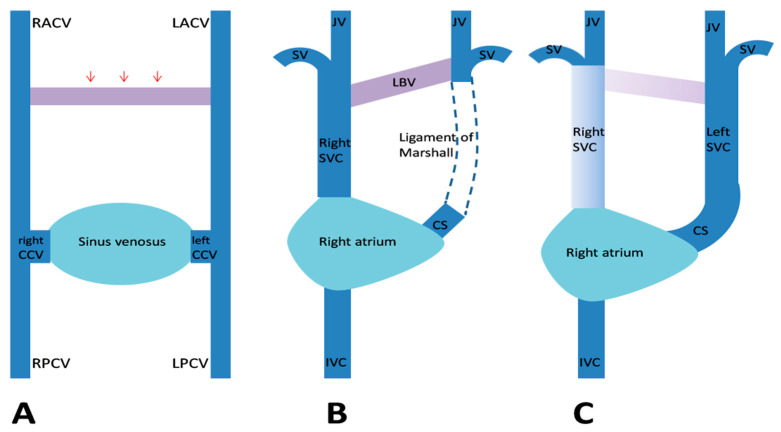
Embryological development of a persistent left superior vena cava (SVC). (**A**) The venous drainage system of the embryo, with transverse anastomosis (red arrows) forming between the anterior cardinal veins. (**B**) Normal regression of the proximal segment of the left anterior cardinal vein (LACV) and the formation of the ligament of Marshall. (**C**) When this obliteration fails to occur, a left SVC develops. Abbreviations: CCV, common cardinal vein; CS, coronary sinus; IVC, inferior vena cava; JV, internal jugular vein; LBV, left brachiocephalic vein; LPCV, left posterior cardinal vein; RACV, right anterior cardinal vein; RPCV, right posterior cardinal vein; and SV, subclavian vein (Figure modified from Figure 5 in [17]).

**Figure 4:**
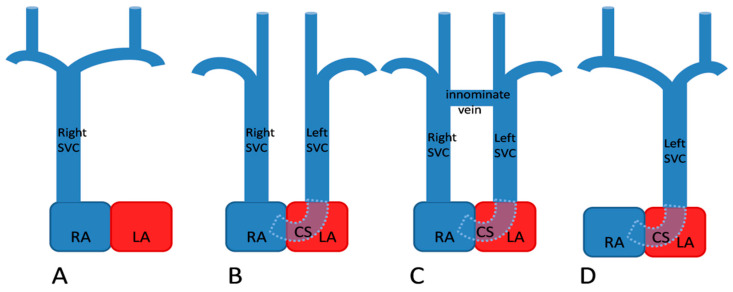
Anatomic types of persistent left superior vena cava (SVC). (**A**) Normal, right-sided SVC (**B**) Double SVC without any anastomosis (**C**) Double SVC with a transverse anastomosis between the two venae cavae (**D**) Left SVC with absent right SVC. Abbreviations: CS, coronary sinus; LA, left atrium; and RA, right atrium (Figure modified from Figure 36.8 in [18]).

By the fifth week of gestation, the venous blood of the embryo is drained by a pair of anterior and a pair of posterior cardinal veins (CVs). The CVs join together on each side and form the right and left common CVs, also called “the ducts of Cuvier,” which further open in the sinus venosus. During the eighth week of gestation, a transverse anastomosis appears between the upper parts of the anterior CVs, which later forms the left brachiocephalic vein (Figure 3A). The segments of the anterior CVs located distally to the anastomosis form the internal jugular veins. The proximal part of the right anterior CV and the corresponding duct of Cuvier form the normal right SVC. The left duct of Cuvier develops into the coronary sinus and the oblique vein, while the proximal segment of the left anterior CV normally regresses and forms the ligament of Marshall (Figure 3B). When this obliteration fails to occur, a left SVC develops that will drain in the right atrium by the coronary sinus (Figure 3C) [1,15,16]. In 10–20% of cases, the proximal part of the right anterior CV regresses, resulting in the absence of the right SVC and a unique PLSVC (Figure 4).

### 3.2. Associated Congenital Anomalies

A PLSVC—particularly the isolated type—is associated with other congenital cardiac defects in up to 46% of cases [3]. The most frequently reported congenital cardiac defects are atrial and ventricular septal defects, bicuspid aortic valves, the coarctation of the aorta, coronary sinus atresia, anomalous pulmonary venous return, and tetralogy of Fallot [19]. Complex malformations, such as Shone’s syndrome, and systemic anomalies, such as VACTERL association and esophageal atresia, have also been reported [5,11].

Subaortic stenosis associated with an isolated PLSVC diagnosed in previously healthy adults is a less frequent finding. While it is usually considered a congenital malformation, later studies have supported the idea that subaortic stenosis is a rather acquired lesion. Apparently, a certain morphology of the left ventricular outflow tract (LVOT), such as a steep aortoseptal angle, would increase the turbulence of the flow in the LVOT, creating endothelial lesions that would further promote fibromuscular proliferation and the development of the subaortic membrane [20]. Agnoletti et al. observed that malformations such as the coarctation of the aorta, subaortic stenosis, and mitral stenosis had a higher incidence in patients with a PLSVC than in patients without this venous anomaly. Therefore, they suggested that a PLSVC can perturb the normal development of the left ventricle and therefore it is associated with the development of the left ventricle inflow and outflow obstructive lesions [21]. Furthermore, it appears that a PLSVC may influence the development of secondary subaortic stenosis after a heart operation for another cardiac anomaly, as was shown by Kalfa et al. in their study [22]. However, discrete subaortic stenosis diagnosed in adulthood has a slow progression over time, and the aortic regurgitation is generally of mild severity, as in the presented case [8].

### 3.3. Practical Implications

A PLSVC should always be suspected when a dilated coronary sinus is observed on transthoracic echocardiography, and an agitated saline contrast study can be used for confirmation [23,24]. When a PLSVC with an absent right SVC is present, the saline contrast injected in a vein from either arm will firstly appear in the coronary sinus and, afterwards, in the right atrium [25]. Transesophageal echocardiography, computed tomography, or magnetic resonance angiography can be performed for a more extensive evaluation of associated cardiac abnormalities and the venous system [26,27].

While a PLSVC draining in the right atrium has no hemodynamic impact, patients may display signs and symptoms caused by the associated cardiac defects or arrhythmic events [5]. The increased arrhythmic risk of a PLSVC is a consequence of alterations in the formation and functionality of the sinus node and the persistence of embryologic pacemaker tissue in the proximity of coronary sinus, with preserved potential to initiate tachyarrhythmias. The dilatation of the coronary sinus may further lead to atrioventricular node stretching and predispose one to reentrant tachycardias or conduction disturbances [28,29,30]. As a consequence, a significant proportion of patients with a PLSVC require permanent pacemaker and/or cardioverter-defibrillator implantation. The transvenous implantation of the leads is particularly difficult when the innominate vein and the right SVC are absent. In this case, the leads are advanced through the left SVC and the coronary sinus. Due to the acute angle between the ostium of the coronary sinus and the tricuspid valve, advancing the ventricular electrode through the tricuspid orifice is technically difficult and may be facilitated by forming a loop in the right atrium or by using a pre-shaped stylet [4,31,32,33,34,35,36]. Other pacing modalities such as leadless pacemaker or epicardial pacing systems might be an option whenever conventional therapy cannot be implemented [33,37]. Cardiovascular surgery in patients with a PLSVC requires special consideration because specific heart cannulation techniques are needed and retrograde cardioplegia may lead to insufficient myocardial protection [38,39]. In addition, the ligation of an isolated PLSVC may cause SVC syndrome and myocardial ischemia if coronary sinus atresia is associated [5,40].

## 4. Conclusions

An isolated persistent left superior vena cava associated with discrete subaortic stenosis is an extremely rare finding. Whenever diagnosing one of these anomalies, one should carefully evaluate the presence of other congenital cardiovascular defects. The visualization of a dilated coronary sinus on transthoracic echocardiography should raise awareness of this venous anomaly, and an agitated saline contrast study should be taken into consideration as a simple, non-invasive method of diagnosis. Physicians performing intracardiac device placement should be familiar with its anatomic variants and with the technical alternatives to overcome intraprocedural difficulties.

## Supplementary Materials

The following are available online at https://www.mdpi.com/2075-4418/10/10/847/s1, Figure S1: Doppler evaluation of the subaortic obstruction. Figure S2: Fluoroscopic image of the final trajectory of the pacemaker lead. Video S1: Transthoracic echocardiography, apical 3-chamber view, showing the subaortic membrane and the dilated coronary sinus. Video S2: Transthoracic echocardiography, apical 3-chamber view, showing the mild aortic regurgitation. Video S3: Transthoracic echocardiography, apical 4-chamber view, showing the cardiac cavities with normal dimensions and the left ventricle with normal systolic function. Video S4: Transesophageal echocardiography view showing the subaortic membrane and the systolic fluttering of the aortic valve. Video S5: Venography performed from left subclavian access, demonstrating a left SVC draining in the right atrium via the coronary sinus but without the possibility to clearly assess the status of the right SVC. Video S6: Venography performed from the right subclavian access demonstrating the absence of the SVC on the right side.
