# Supplementary figures and images for: Persistent Left Superior Vena Cava with Absent Right Superior Vena Cava and Discrete Subaortic Stenosis Diagnosed in a Patient with Sick Sinus Syndrome: A Case Report and Brief Review of the Literature
Source: Diagnostics (Basel). 2020 Oct 19;10(10):847. doi: 10.3390/diagnostics10100847 (PMC7589949; doi:10.3390/diagnostics10100847)

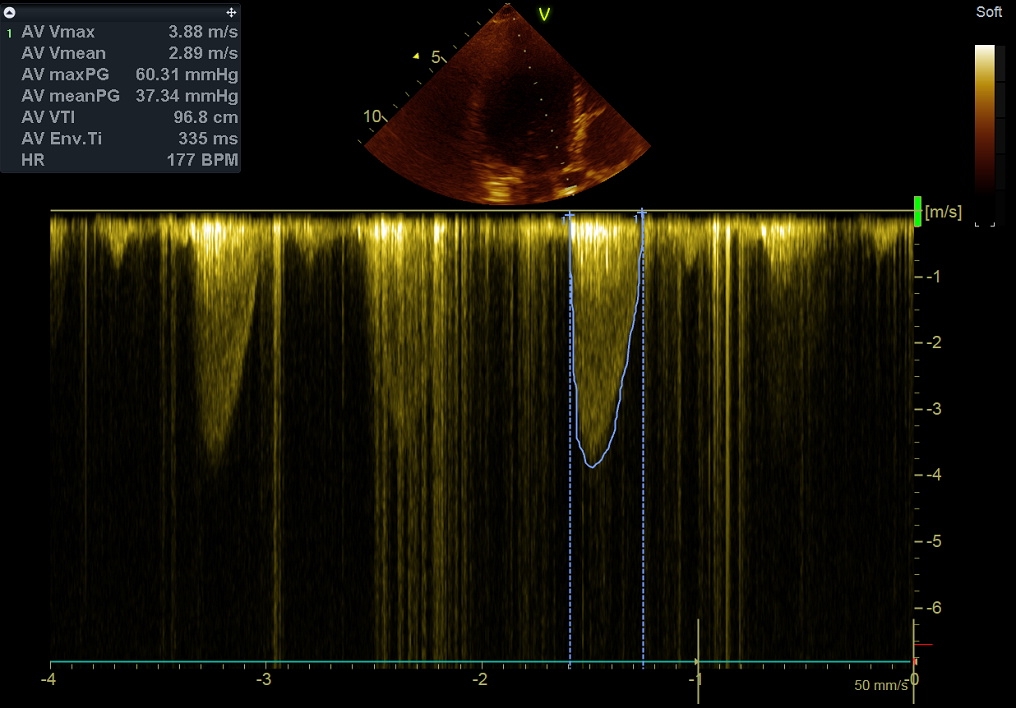

Supplement: Supplementary file 1 [file diagnostics-10-00847-s001.zip › Figure S1.jpg]

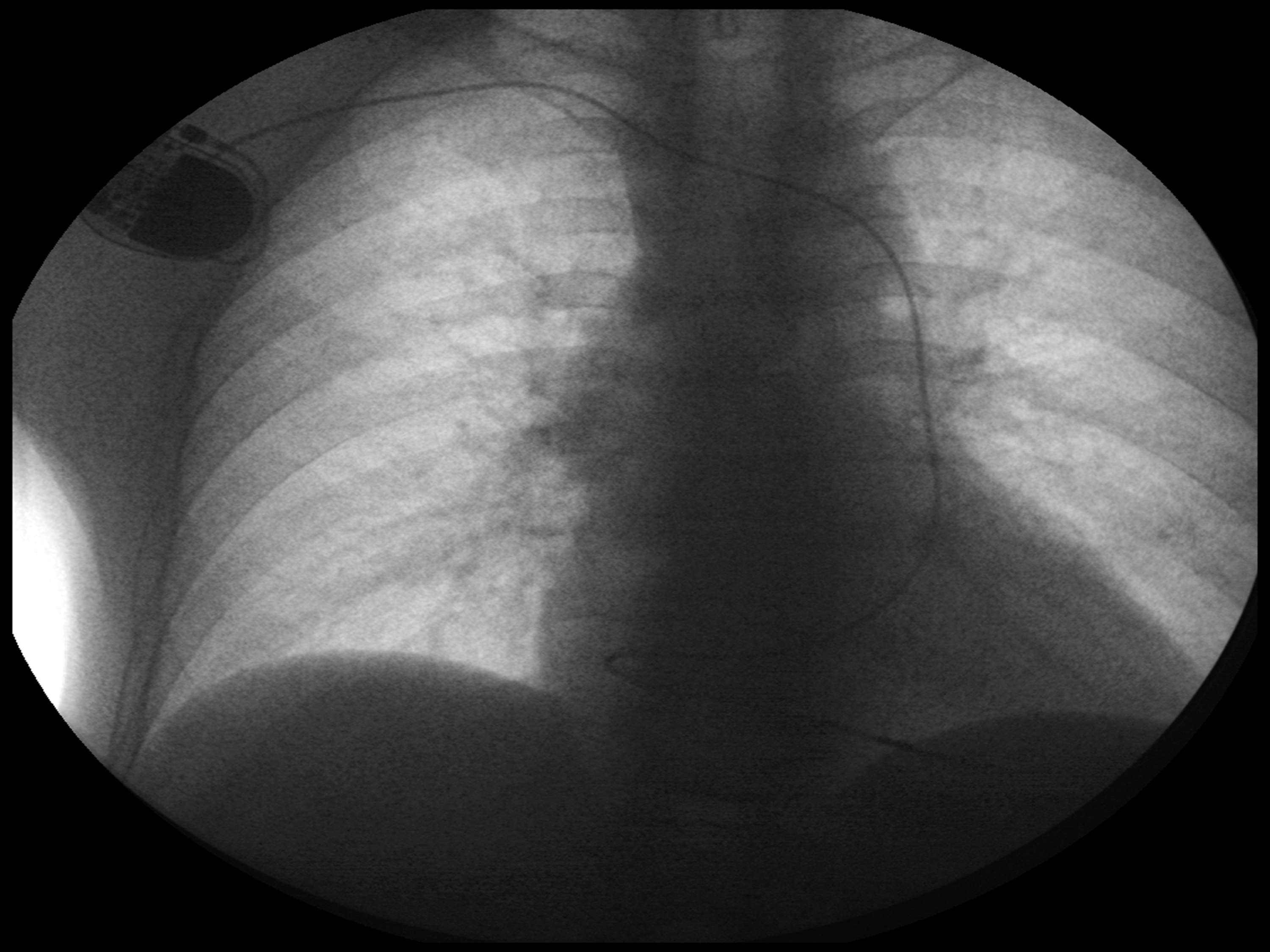

Supplement: Supplementary file 1 [file diagnostics-10-00847-s001.zip › Figure S2.PNG]
